# Mapping the Prevalence of Physical Inactivity in U.S. States, 1984-2015

**DOI:** 10.1371/journal.pone.0168175

**Published:** 2016-12-13

**Authors:** Ruopeng An, Xiaoling Xiang, Yan Yang, Hai Yan

**Affiliations:** 1 Department of Kinesiology and Community Health, College of Applied Health Sciences, University of Illinois at Urbana-Champaign, Champaign, Illinois, United States of America; 2 Feinburg School of Medicine, Northwestern University, Chicago, Illinois, United States of America; Leibniz Institute for Prevention Research and Epidemiology BIPS, GERMANY

## Abstract

**Background:**

Physical inactivity is a leading cause of morbidity, disability and premature mortality in the U.S. and worldwide. This study aimed to map the prevalence of physical inactivity across U.S. states over the past three decades, and estimate the over-time adjusted changes in the prevalence of physical inactivity in each state.

**Methods:**

Individual-level data (N = 6,701,954) were taken from the 1984–2015 Behavioral Risk Factor Surveillance System (BRFSS), an annually repeated cross-sectional survey of state-representative adult population. Prevalence of self-reported leisure-time physical inactivity was estimated by state and survey year, accounting for the BRFSS sampling design. Logistic regressions were performed to estimate the changes in the prevalence of physical inactivity over the study period for each state, adjusting for individual characteristics including sex, age, race/ethnicity, education, marital status, and employment status.

**Results:**

The prevalence of leisure-time physical inactivity varied substantially across states and survey years. In general, the adjusted prevalence of physical inactivity gradually declined over the past three decades in a majority of states. However, a substantial proportion of American adults remain physically inactive. Among the 50 states and District of Columbia, 45 had over a fifth of their adult population without any leisure-time physical activity, and 8 had over 30% without physical activity in 2015. Moreover, the adjusted prevalence of physical inactivity in several states (Arizona, North Carolina, North Dakota, Utah, West Virginia, and Wyoming) remained largely unchanged or even increased (Minnesota and Ohio) over the study period.

**Conclusions:**

Although the prevalence of physical inactivity declined over the past three decades in a majority of states, the rates remain substantially high and vary considerably across states. Closely monitoring and tracking physical activity level using the state physical activity maps can help guide policy and program development to promote physical activity and reduce the burden of chronic disease.

## Introduction

Physical inactivity is a leading cause of morbidity, disability and premature mortality in the U.S. and worldwide [1]. Promoting physical activity has long been a public health priority [2]. However, 4 in 5 U.S. adults do not meet recommended levels of physical activity guidelines [3]. Various programs such as provision of economic incentives [4], mass media campaigns [5], point-of-decision prompts [6], neighborhood built environment remodeling [7], social network interventions [8], etc., have been employed to promote active living. However, existing programs addressing sedentary behavior and physical inactivity have only limited success in facilitating long-term behavior modification and maintenance [9, 10].

State-level public health departments typically monitor outbreaks of infectious diseases and prevalence/incidence of key adverse health outcomes such as all-cause and disease-specific mortality, cancer, cardiovascular disease, diabetes, and obesity, whereas less attention has been paid to track health behaviors that strongly correlate with major disease onset including dietary intake, physical activity, and sedentary behavior [11–14]. This information gap to some extent compromises state departments’ capacity to identify the underlining contributors to a disease epidemic, predict future disease burden, optimize resource allocation, and design/implement tailored and targeted interventions to address risk factors. Closely monitoring the state prevalence of physical inactivity can be essential in informing policy makers and various stakeholders and helping shape public health policies in an effort to promote a more active lifestyle. Toward this aim, utilization of state-representative data with physical inactivity measure is warranted, and the optimal data source needs to satisfy two criteria: repeatedly collected over a long period of time to allow trajectory mapping, and covering all states in the U.S. with the same measuring instrument to facilitate cross-state comparison.

The obesity prevalence maps, available at the Centers for Disease Control and Prevention (CDC) web portal (https://www.cdc.gov/obesity/data/prevalence-maps.html), were created based on data collected by individual U.S. states that adopted the Behavioral Risk Factor Surveillance System (BRFSS). These obesity prevalence maps remain highly influential over the past decade, and have been widely cited in both scientific literature and mess media [15–17]. The underlining importance of these maps has been extended from tracking, documenting, sharing, and contrasting obesity prevalence across U.S. states over time to warning, inspiring, and stimulating public awareness and societal actions. By the same token, the aims of this study were to (1) estimate and map the prevalence of physical inactivity across U.S. states over the last 32 years from 1984 to 2015 based on data from the BRFSS, and (2) estimate and track the adjusted changes in the prevalence of physical inactivity in each state during the BRFSS survey period. The exhibits (i.e., maps, figures, and tables) shown in this study could be used by various entities to help understand and delineate the regional and temporal variations in the prevalence of physical inactivity in the U.S., and inspire and motivate policy and social changes that promote active living.

## Methods

### Participants

Individual-level data came from the BRFSS 1984–2015 surveys. The BRFSS is a state-based system of annually repeated cross-sectional telephone surveys that collect information on health risk behaviors, preventive health practices, and health care access primarily related to chronic disease and injury. During 1984–2010, the BRFSS was administered over landline telephones alone; whereas since 2011, refinement to the BRFSS sampling schemes has been made to include data received from cell phone users as well, which facilitates the inclusion of a broader demographic and provides a more comprehensive reflection of the nation’s health status.

The BRFSS questionnaire was developed in collaboration between CDC and public health departments in each of the states and the District of Columbia. In order to maintain consistency across states, the BRFSS sets standard protocols for data collection. These standards allow for cross-state data comparison. States may opt to contract with a private company or university to conduct interviews or conduct interviews internally, but regardless of who conducts data collection, it is conducted according to the BRFSS protocols. States may determine that they would like to sample by county, public health district or other sub-state geography in order to make comparisons of geographic areas within their states. The response rate averaged approximately 40%-50% across states and years. Sampling weights were constructed to account for survey non-response. Detailed information about the BRFSS including questionnaires, sampling design and survey datasets can be found on its web portal (http://www.cdc.gov/brfss/annual_data/annual_data.htm).

Among a total of 7,274,451 adults 18 years of age and above who participated in the BRFSS 1984–2015 surveys, the following individuals were excluded from the analyses: missing data on self-reported leisure-time physical activity (mostly due to the relevant question being moved from the core questionnaire to the optional module that was administered only in a few states in the BRFSS 1993, 1995, 1997, and 1999), 512,637; and missing data on other individual characteristics (i.e., sex, age, race/ethnicity, education, marital status, or employment status), 59,860. The remaining 6,701,954 survey participants were included in the final sample.

As a sensitivity analysis, we compared individual characteristics (i.e., sex, age, race/ethnicity, education, marital status, and employment status) between those who reported leisure-time physical activity and those who did not report leisure-time physical activity due to non-adoption of the optional module within the same state in consecutive years (e.g., California in 1992 vs. 1993). Two-sample t-tests were performed on continuous variable age, and chi-squared tests were performed on other dichotomous/categorical variables including sex, race/ethnicity, education, marital status, and employment status. Bonferroni corrections were implemented to adjust for multiple comparisons. A majority of test statistics were statistically nonsignificant at *p*-value < 0.01, and in general the quantitative differences between samples from two consecutive years within the same state were rather small. Given results from this sensitivity analysis and the small proportion of missing value due to optional module administration over the entire sample (7%), it is unlikely to substantially affect our main findings regarding physical inactivity prevalence and trajectory.

### Measure of leisure-time physical inactivity

The Physical Activity Rotating Core (PARC) has been an integral part of the BRFSS since 1984 [18]. One major use of the PARC is to monitor the percentage of the U.S. adult population meeting physical activity guidelines [18]. The guidelines highlight the importance of avoiding physical inactivity—even low amounts of physical activity reduce the risk of premature mortality, and the most dramatic difference in mortality risk is found between those who are physically inactive and those with low levels of activity [19]. The umbrella (first) question of the PARC, which serves as the outcome of our interest, slightly changed from 2001 onwards. In the BRFSS 1984–2000 surveys, the question reads, “The next few questions are about exercise, recreation, or physical activities other than your regular job duties. During the past month, did you participate in any physical activities or exercises such as running, calisthenics, golf, gardening, or walking for exercise?” In the BRFSS 2001–2015 surveys, the question reads, “During the past month, other than your regular job, did you participate in any physical activities or exercises such as running, calisthenics, golf, gardening, or walking for exercise?” Self-reported leisure-time physical inactivity was ascertained from answers of “no” to the relevant questions in the BRFSS 1984–2000 and 2001–2015 surveys. This modification in question wording was primary due to the switch from asking exercise frequency and type (during 1984–2000) to soliciting more detailed information regarding exercise intensity (moderate or vigorous) and duration (from 2001 and onward).

### Residential state

Not all U.S. states participated in each BRFSS survey over the past 32 years from 1984 to 2015. Moreover, questions on leisure-time physical activity were moved from the core questionnaire (which was administered in all states) to the optional module (which was administered in a small number of opt-in states) in 1993, 1995, 1997, and 1999. Table 1 lists the states that participated in each BRFSS survey during 1984–2015.

**Table 1:** List of U.S. states that participated in BRFSS 1984–2015 and administered physical activity questionnaire.

BRFSS	Participating states
1984	AZ CA ID IL IN MN MT NC OH RI SC TN UT WV WI
1985	AZ CA CT DC FL GA ID IL IN KY MN MT NY NC ND OH RI SC TN UT WV WI
1986	AL AZ CA DC FL GA HI ID IL IN KY MA MN MO MT NM NY NC ND OH RI SC TN UT WV WI
1987	AL AZ CA DC FL GA HI ID IL IN KY ME MD MA MN MO MT NE NH NM NY NC ND OH RISC SD TN TX UT WA WV WI
1988	AL AZ CA CT DC FL GA HI ID IL IN IA KY ME MD MA MI MN MO MT NE NH NM NY NC ND OH OK RI SC SD TN TX UT WA WV WI
1989	AL AZ CA CT DC FL GA HI ID IL IN IA KY ME MD MA MI MN MO MT NE NH NM NY NC ND OH OK OR PA RI SC SD TN TX UT VA WA WV WI
1990	AL AZ CA CO CT DE DC FL GA HI ID IL IN IA KY LA ME MD MA MI MN MS MO MT NE NH NM NY NC ND OH OK OR PA RI SC SD TN TX UT VT VA WA WV WI
1991	AL AK AZ AR CA CO CT DE DC FL GA HI ID IL IN IA KY LA ME MD MA MI MN MS MO MT NE NH NJ NM NY NC ND OH OK OR PA RI SC SD TN TX UT VT VA WA WV WI
1992	AL AK AZ CA CO CT DE DC FL GA HI ID IL IN IA KS KY LA ME MD MA MI MN MS MO MT NE NV NH NJ NM NY NC ND OH OK OR PA RI SC SD TN TX UT VT VA WA WV WI
1993	AZ DC KS ME MT PA SC TN VA
1994	AL AK AZ AR CA CO CT DE DC FL GA HI ID IL IN IA KS KY LA ME MD MA MI MN MS MO MT NE NV NH NJ NM NY NC ND OH OK OR PA SC SD TN TX UT VT VA WA WV WI WY
1995	AZ CA IL KS NJ OK PA SC SD VA WY
1996	AL AK AZ AR CA CO CT DE DC FL GA HI ID IL IN IA KS KY LA ME MD MA MI MN MS MO MT NE NV NH NJ NM NY NC ND OH OK OR PA RI SC SD TN TX UT VT VA WA WV WI WY
1997	AZ IL IA KY NJ NY OH OK SC TN VA WY
1998	AL AK AZ AR CA CO CT DE DC FL GA HI ID IL IN IA KS KY LA ME MD MA MI MN MS MO MT NE NV NH NJ NM NY NC ND OH OK OR PA RI SC SD TN TX UT VT VA WA WV WI WY
1999	GA HI IL MI NE NM OH OK TN UT VA
2000	AL AK AZ AR CA CO CT DE DC FL GA HI ID IL IN IA KS KY LA ME MD MA MI MN MS MO MT NE NV NH NJ NM NY NC ND OH OK OR PA RI SC SD TN TX UT VT VA WA WV WI WY
2001	AL AK AZ AR CA CO CT DE DC FL GA HI ID IL IN IA KS KY LA ME MD MA MI MN MS MO MT NE NV NH NJ NM NY NC ND OH OK OR PA RI SC SD TN TX UT VT VA WA WV WI WY
2002	AL AK AZ AR CA CO CT DE DC FL GA HI ID IL IN IA KS KY LA ME MD MA MI MN MS MO MT NE NV NH NJ NM NY NC ND OH OK OR PA RI SC SD TN TX UT VT VA WA WV WI WY
2003	AL AK AZ AR CA CO CT DE DC FL GA HI ID IL IN IA KS KY LA ME MD MA MI MN MS MO MT NE NV NH NJ NM NY NC ND OH OK OR PA RI SC SD TN TX UT VT VA WA WV WI WY
2004	AL AK AZ AR CA CO CT DE DC FL GA ID IL IN IA KS KY LA ME MD MA MI MN MS MO MT NE NV NH NJ NM NY NC ND OH OK OR PA RI SC SD TN TX UT VT VA WA WV WI WY
2005	AL AK AZ AR CA CO CT DE DC FL GA HI ID IL IN IA KS KY LA ME MD MA MI MN MS MO MT NE NV NH NJ NM NY NC ND OH OK OR PA RI SC SD TN TX UT VT VA WA WV WI WY
2006	AL AK AZ AR CA CO CT DE DC FL GA HI ID IL IN IA KS KY LA ME MD MA MI MN MS MO MT NE NV NH NJ NM NY NC ND OH OK OR PA RI SC SD TN TX UT VT VA WA WV WI WY
2007	AL AK AZ AR CA CO CT DE DC FL GA HI ID IL IN IA KS KY LA ME MD MA MI MN MS MO MT NE NV NH NJ NM NY NC ND OH OK OR PA RI SC SD TN TX UT VT VA WA WV WI WY
2008	AL AK AZ AR CA CO CT DE DC FL GA HI ID IL IN IA KS KY LA ME MD MA MI MN MS MO MT NE NV NH NJ NM NY NC ND OH OK OR PA RI SC SD TN TX UT VT VA WA WV WI WY
2009	AL AK AZ AR CA CO CT DE DC FL GA HI ID IL IN IA KS KY LA ME MD MA MI MN MS MO MT NE NV NH NJ NM NY NC ND OH OK OR PA RI SC SD TN TX UT VT VA WA WV WI WY
2010	AL AK AZ AR CA CO CT DE DC FL GA HI ID IL IN IA KS KY LA ME MD MA MI MN MS MO MT NE NV NH NJ NM NY NC ND OH OK OR PA RI SC SD TN TX UT VT VA WA WV WI WY
2011	AL AK AZ AR CA CO CT DE DC FL GA HI ID IL IN IA KS KY LA ME MD MA MI MN MS MO MT NE NV NH NJ NM NY NC ND OH OK OR PA RI SC SD TN TX UT VT VA WA WV WI WY
2012	AL AK AZ AR CA CO CT DE DC FL GA HI ID IL IN IA KS KY LA ME MD MA MI MN MS MO MT NE NV NH NJ NM NY NC ND OH OK OR PA RI SC SD TN TX UT VT VA WA WV WI WY
2013	AL AK AZ AR CA CO CT DE DC FL GA HI ID IL IN IA KS KY LA ME MD MA MI MN MS MO MT NE NV NH NJ NM NY NC ND OH OK OR PA RI SC SD TN TX UT VT VA WA WV WI WY
2014	AL AK AZ AR CA CO CT DE DC FL GA HI ID IL IN IA KS KY LA ME MD MA MI MN MS MO MT NE NV NH NJ NM NY NC ND OH OK OR PA RI SC SD TN TX UT VT VA WA WV WI WY
2015	AL AK AZ AR CA CO CT DE DC FL GA HI ID IL IN IA KS KY LA ME MD MA MI MN MS MO MT NE NV NH NJ NM NY NC ND OH OK OR PA RI SC SD TN TX UT VT VA WA WV WI WY

### Individual characteristics

Various individual characteristics have been linked to physical activity, such as sex [20], age [21], race/ethnicity [22], education level [23], employment status [24], and marital status [25]. Temporal changes in the distributions of these individual characteristics among the state population may confound in the estimated prevalence of physical inactivity. Therefore, we controlled these individual characteristics in logistic regressions: a dichotomous variable for female (male as the reference group), 2 continuous variables for age in years and age in years squared (to account for potential nonlinear relationship between leisure-time physical inactivity and age), 4 categorical variables for race/ethnicity (non-Hispanic black, non-Hispanic Asian or Pacific Islander, non-Hispanic other race or multi-race, and Hispanic, with non-Hispanic white as the reference group), 4 categorical variables for education attainment (some high school, high school graduate or equivalent, some college or equivalent, and college graduate or higher, with primary school or lower as the reference group), 6 categorical variables for employment status (unemployed for a year or less, unemployed for over a year, homemaker, student, retired, and unable to work, with employed as the reference group), and 2 categorical variables for marital status (divorced or widowed or separated, and never married, with married as the reference group).

### Statistical analyses

Prevalence of leisure-time physical inactivity was estimated for each U.S. state and survey year, accounting for the BRFSS sampling design. Based on its empirical distribution, the estimated state- and year-specific prevalence of leisure-time physical inactivity was classified into 5 categories—lower than 25%, 25% to less than 30%, 30% to less than 35%, 35% to less than 40%, and 40% or higher.

Separate logistic regressions were conducted to estimate the changes in the prevalence of leisure-time physical inactivity over the BRFSS survey period for each U.S. state, adjusting for individual characteristics (i.e., sex, age, race/ethnicity, education, employment status, and marital status). The key independent variables were a series of categorical variables for survey years, with the first survey year as the reference group (i.e., baseline). For instance, Virginia was surveyed by the BRFSS regarding leisure-time physical activity during 1989–2015, and thus had 26 categorical variables denoting each survey year from 1990 to 2015, with the survey year of 1989 as the baseline. Average marginal effects were calculated based on the estimated coefficients from logistical regressions. The use of average marginal effect converted odds ratio to change in probability (i.e., prevalence) relative to the baseline.

All statistical analyses were conducted using Stata 14.2 SE version (StataCorp, College Station, TX). The BRFSS sampling design was accounted for in both descriptive statistics and regression analyses. More specifically, Stata’s survey functions “svy” were used to incorporate BRFSS sampling strata, primary sampling unit, and sampling weight in estimation. Average marginal effects were calculated using the Stata function “margins”.

### Human subject protection

The BRFSS was approved by the National Center for Health Statistics Research Ethics Review Board. This study used the BRFSS de-identified public data and was deemed exempt from human subjects review by the University of Illinois at Urbana-Champaign Institutional Review Board.

## Results

**Fig 1:**
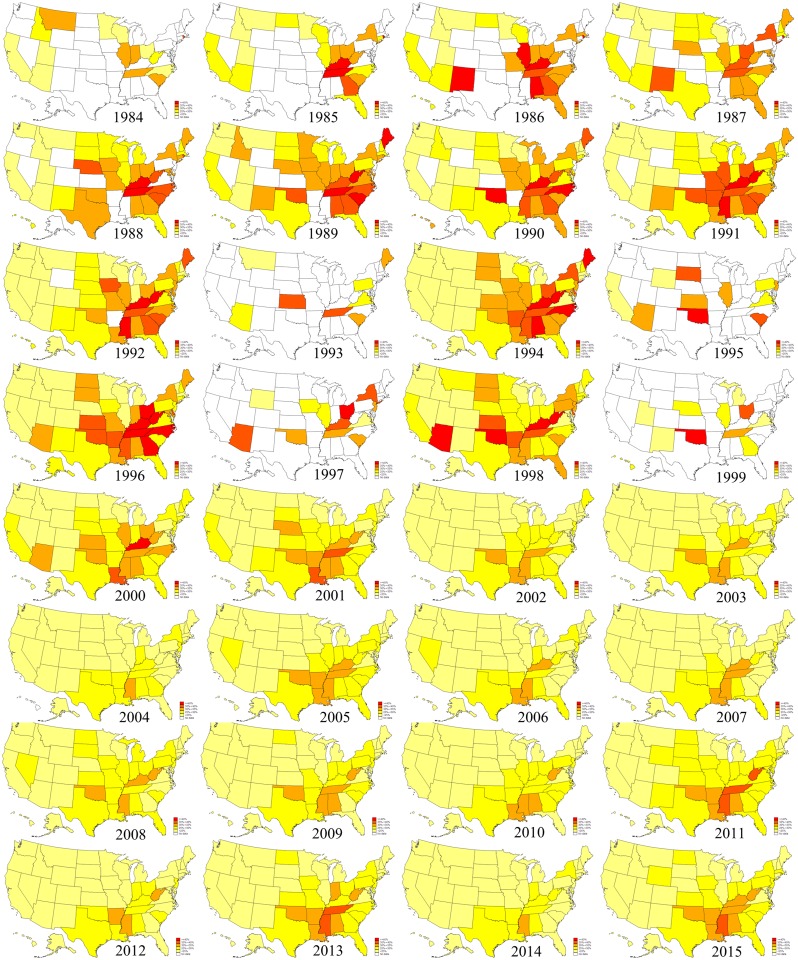
Prevalence of leisure-time physical inactivity among U.S. states, 1984–2015.

Fig 1 illustrates the prevalence of leisure-time physical inactivity in U.S. states by survey year from 1984 to 2015. In general, the prevalence of leisure-time physical inactivity declined over the survey period. During 1988–1998, 8–12 states had a prevalence of 35% or higher, whereas during 2005–2015, only 0–3 states had a prevalence of 35% or higher. There were substantial disparities in the prevalence of leisure-time physical inactivity both across states and survey years. For example, in 1990, the prevalence of leisure-time physical inactivity was 51.9% in the District of Columbia, nearly three folds of that (18.0%) in Montana. In 1996, the prevalence of leisure-time physical inactivity was 51.3% in Georgia, over three folds of that (17.0%) in Utah. The prevalence of leisure-time physical inactivity in DC increased from 38.4% in 1984 to 51.9% in 1990, and then gradually declined to 18.8% in 2015. The prevalence of leisure-time physical inactivity in Arizona increased from 20.4% in 1984 to 51.3% in 1998, and then gradually declined to its survey-inception level (24.4%) in 2015. Tables 2–7 report point estimates and corresponding 95% confidence intervals for the prevalence of leisure-time physical inactivity in each U.S. state by survey year.

**Table 2:** Prevalence (%) of leisure-time physical inactivity in U.S. states, 1984–1989.

State	1984	1985	1986	1987	1988	1989
**AK**						
**AL**			43.39 (38.45, 48.48)	32.83 (30.08, 35.71)	31.30 (28.65, 34.07)	35.40 (33.01, 37.86)
**AR**						
**AZ**	20.35 (17.48, 23.57)	27.91 (25.15, 30.86)	25.66 (22.89, 28.65)	27.53 (24.98, 30.24)	24.22 (21.60, 27.06)	24.01 (21.56, 26.64)
**CA**	23.85 (20.95, 27.02)	28.00 (25.37, 30.79)	27.25 (24.77, 29.88)	26.12 (23.82, 28.55)	22.32 (20.38, 24.40)	25.39 (23.26, 27.65)
**CO**						
**CT**		29.85 (26.76, 33.13)			33.52 (30.41, 36.79)	32.28 (29.47, 35.22)
**DC**		38.42 (33.88, 43.16)	43.93 (40.55, 47.36)	38.70 (35.36, 42.15)	48.05 (44.64, 51.48)	49.27 (46.29, 52.26)
**DE**						
**FL**		28.43 (25.00, 32.12)	31.16 (28.13, 34.35)	30.71 (27.76, 33.83)	26.08 (23.62, 28.70)	25.65 (23.37, 28.08)
**GA**		38.51 (34.76, 42.40)	38.04 (34.96, 41.21)	33.35 (30.58, 36.24)	32.19 (29.00, 35.55)	36.69 (34.10, 39.37)
**HI**			29.92 (26.93, 33.10)	25.58 (22.96, 28.39)	25.76 (23.23, 28.46)	27.97 (25.59, 30.48)
**IA**					31.05 (27.38, 34.96)	34.11 (31.22, 37.13)
**ID**	28.43 (24.28, 32.99)	23.58 (20.46, 27.02)	19.91 (17.29, 22.81)	22.05 (19.84, 24.42)	24.65 (22.42, 27.02)	30.56 (28.15, 33.08)
**IL**	31.78 (27.95, 35.87)	29.98 (26.88, 33.27)	40.32 (37.12, 43.61)	31.91 (29.50, 34.41)	27.36 (25.04, 29.82)	32.50 (30.11, 34.99)
**IN**	30.23 (25.54, 35.38)	30.90 (27.98, 33.97)	31.92 (29.17, 34.81)	29.15 (27.06, 31.34)	31.39 (29.11, 33.77)	31.30 (29.10, 33.59)
**KS**						
**KY**		41.61 (37.88, 45.44)	45.88 (42.56, 49.23)	39.45 (36.95, 42.01)	40.02 (37.47, 42.63)	39.11 (36.50, 41.79)
**LA**						
**MA**			30.65 (27.58, 33.89)	27.35 (24.75, 30.11)	28.64 (25.98, 31.44)	25.69 (23.01, 28.56)
**MD**				30.93 (27.65, 34.42)	32.94 (29.72, 36.34)	32.18 (29.66, 34.81)
**ME**				33.45 (30.60, 36.43)	35.00 (32.13, 37.98)	42.21 (39.12, 45.36)
**MI**					27.48 (24.72, 30.42)	29.68 (27.72, 31.72)
**MN**	14.60 (12.68, 16.75)	20.81 (19.01, 22.74)	24.72 (23.03, 26.49)	25.85 (24.19, 27.58)	25.86 (24.25, 27.53)	30.96 (29.27, 32.71)
**MO**			34.41 (30.89, 38.11)	29.97 (27.31, 32.77)	30.67 (28.03, 33.44)	31.59 (28.97, 34.33)
**MS**						
**MT**	34.78 (30.94, 38.82)	15.93 (13.64, 18.52)	13.69 (11.65, 16.02)	14.02 (11.94, 16.38)	14.21 (12.19, 16.49)	16.24 (14.07, 18.66)
**NC**	24.58 (21.93, 27.44)	29.12 (26.58, 31.80)	31.48 (28.94, 34.13)	32.02 (29.74, 34.38)	38.54 (35.67, 41.49)	38.24 (35.53, 41.03)
**ND**		25.87 (22.11, 30.03)	29.14 (26.22, 32.24)	26.47 (24.12, 28.95)	25.91 (23.64, 28.31)	27.42 (25.06, 29.92)
**NE**				32.40 (29.51, 35.42)	37.40 (34.62, 40.26)	33.34 (30.78, 36.00)
**NH**				28.53 (25.95, 31.27)	28.77 (25.90, 31.83)	25.38 (22.93, 27.98)
**NJ**						
**NM**			44.84 (41.45, 48.29)	36.75 (33.42, 40.21)	27.02 (24.12, 30.12)	31.22 (28.41, 34.19)
**NV**						
**NY**		33.38 (30.29, 36.63)	34.63 (31.37, 38.03)	38.26 (35.13, 41.50)	33.46 (30.39, 36.67)	33.83 (30.76, 37.05)
**OH**	18.94 (15.77, 22.57)	33.46 (30.30, 36.76)	34.67 (31.61, 37.86)	39.06 (36.34, 41.85)	33.19 (30.42, 36.09)	34.38 (31.61, 37.26)
**OK**					30.67 (27.55, 33.98)	39.90 (36.65, 43.25)
**OR**						17.30 (15.47, 19.29)
**PA**						27.52 (25.23, 29.93)
**RI**	38.68 (35.05, 42.43)	47.08 (43.87, 50.31)	47.09 (44.18, 50.02)	46.35 (43.71, 49.00)	38.39 (35.97, 40.87)	34.34 (31.95, 36.81)
**SC**	30.81 (26.72, 35.22)	33.14 (30.06, 36.38)	30.04 (27.71, 32.49)	29.37 (27.05, 31.79)	37.91 (35.36, 40.52)	41.84 (39.22, 44.52)
**SD**				28.85 (26.13, 31.73)	28.81 (25.93, 31.87)	28.22 (25.97, 30.59)
**TN**	31.38 (27.52, 35.52)	45.08 (41.83, 48.36)	36.96 (34.32, 39.67)	38.85 (36.68, 41.07)	42.83 (40.54, 45.15)	41.77 (39.50, 44.08)
**TX**				29.98 (26.93, 33.23)	33.50 (30.41, 36.74)	27.28 (24.77, 29.95)
**UT**	16.55 (13.68, 19.88)	17.36 (14.93, 20.09)	16.53 (14.30, 19.03)	22.98 (20.55, 25.62)	24.98 (22.37, 27.78)	22.10 (19.95, 24.42)
**VA**						30.14 (27.40, 33.03)
**VT**						
**WA**				19.65 (17.35, 22.18)	20.68 (18.36, 23.20)	21.54 (19.41, 23.83)
**WI**	23.94 (20.52, 27.73)	25.93 (23.06, 29.02)	27.17 (24.61, 29.89)	23.95 (21.53, 26.56)	26.40 (23.69, 29.31)	28.38 (25.76, 31.15)
**WV**	27.96 (24.02, 32.27)	34.94 (31.90, 38.12)	31.94 (29.20, 34.80)	32.70 (30.18, 35.32)	37.73 (35.20, 40.32)	42.47 (39.65, 45.35)
**WY**						

**Table 3:** Prevalence (%) of leisure-time physical inactivity in U.S. states, 1990–1995.

State	1990	1991	1992	1993	1994	1995
**AK**		22.17 (19.17, 25.49)	22.52 (19.53, 25.82)		22.81 (19.94, 25.97)	
**AL**	33.56 (31.31, 35.89)	34.24 (31.91, 36.65)	31.46 (29.24, 33.76)		45.80 (43.10, 48.53)	
**AR**		36.07 (33.07, 39.18)			35.15 (32.56, 37.83)	
**AZ**	20.67 (18.50, 23.01)	24.22 (21.90, 26.70)	24.61 (22.04, 27.37)	25.32 (22.52, 28.35)	23.74 (21.20, 26.48)	33.83 (30.45, 37.39)
**CA**	24.43 (22.50, 26.47)	23.24 (21.56, 25.01)	22.54 (21.13, 24.01)		21.78 (20.32, 23.32)	22.69 (20.84, 24.64)
**CO**	19.44 (17.45, 21.61)	18.67 (16.80, 20.70)	17.16 (15.28, 19.23)		17.16 (15.18, 19.33)	
**CT**	25.47 (23.30, 27.78)	25.65 (23.44, 27.99)	29.81 (27.53, 32.20)		21.96 (19.93, 24.12)	
**DC**	51.87 (48.79, 54.95)	39.52 (36.42, 42.69)	38.26 (35.33, 41.29)	47.95 (44.99, 50.93)	48.56 (45.27, 51.87)	
**DE**	27.45 (25.00, 30.04)	31.37 (28.77, 34.10)	31.16 (28.55, 33.89)		36.35 (34.02, 38.75)	
**FL**	32.16 (29.94, 34.46)	28.51 (26.36, 30.77)	26.87 (25.05, 28.77)		27.92 (26.27, 29.63)	
**GA**	36.95 (34.51, 39.47)	39.84 (37.23, 42.51)	39.58 (37.03, 42.18)		32.96 (30.71, 35.30)	
**HI**	31.58 (29.04, 34.24)	23.32 (21.20, 25.59)	26.31 (23.85, 28.93)		20.73 (18.74, 22.87)	
**IA**	33.76 (31.05, 36.57)	29.96 (27.50, 32.55)	36.15 (33.71, 38.65)		33.21 (31.23, 35.25)	
**ID**	27.78 (25.43, 30.26)	22.00 (19.84, 24.32)	20.27 (18.18, 22.53)		21.86 (19.73, 24.15)	
**IL**	32.05 (29.57, 34.64)	36.09 (33.73, 38.52)	33.03 (30.85, 35.28)		33.44 (31.09, 35.88)	32.42 (29.68, 35.29)
**IN**	27.47 (25.50, 29.53)	26.90 (24.85, 29.06)	24.62 (22.70, 26.64)		29.62 (27.63, 31.68)	
**KS**			28.92 (26.44, 31.53)	38.10 (35.41, 40.87)	34.39 (31.75, 37.12)	30.89 (28.68, 33.19)
**KY**	41.86 (39.36, 44.40)	42.06 (39.50, 44.66)	41.56 (39.21, 43.96)		45.85 (43.54, 48.17)	
**LA**	28.67 (25.30, 32.29)	32.50 (29.99, 35.12)	34.86 (32.22, 37.60)		33.50 (30.84, 36.27)	
**MA**	23.32 (20.74, 26.10)	25.25 (22.81, 27.86)	21.83 (19.60, 24.23)		24.00 (21.80, 26.34)	
**MD**	30.36 (27.73, 33.12)	27.64 (25.23, 30.18)	32.28 (29.97, 34.69)		30.14 (28.54, 31.79)	
**ME**	36.32 (33.38, 39.36)	34.86 (32.06, 37.76)	36.80 (33.88, 39.81)	34.69 (31.72, 37.79)	40.73 (37.87, 43.66)	
**MI**	32.37 (30.30, 34.52)	28.60 (26.63, 30.66)	24.55 (22.66, 26.54)		23.03 (21.21, 24.96)	
**MN**	24.88 (23.28, 26.56)	23.68 (22.10, 25.32)	18.74 (17.31, 20.25)		21.72 (20.37, 23.14)	
**MO**	32.65 (30.08, 35.32)	36.23 (33.47, 39.08)	32.30 (29.80, 34.90)		31.76 (29.10, 34.54)	
**MS**	39.23 (36.33, 42.22)	42.56 (39.82, 45.35)	48.00 (45.24, 50.78)		38.45 (35.64, 41.34)	
**MT**	17.99 (15.81, 20.38)	16.64 (14.41, 19.15)	17.92 (15.66, 20.43)	19.41 (17.07, 21.98)	21.02 (18.69, 23.54)	
**NC**	40.43 (38.04, 42.86)	33.48 (31.00, 36.04)	34.18 (31.92, 36.50)		42.77 (40.33, 45.24)	
**ND**	26.86 (24.53, 29.31)	27.98 (25.77, 30.31)	27.05 (24.75, 29.47)		31.98 (29.67, 34.38)	
**NE**	24.91 (22.65, 27.33)	25.10 (22.68, 27.70)	26.88 (24.62, 29.26)		24.31 (22.23, 26.51)	
**NH**	19.67 (17.58, 21.94)	21.09 (18.93, 23.43)	23.79 (21.45, 26.30)		25.62 (23.21, 28.18)	
**NJ**		31.42 (28.65, 34.33)	32.10 (29.45, 34.88)		30.77 (28.11, 33.56)	30.40 (27.45, 33.52)
**NM**	28.03 (25.16, 31.08)	33.72 (30.85, 36.72)	29.56 (26.61, 32.70)		19.45 (17.14, 21.99)	
**NV**			23.48 (21.12, 26.03)		21.55 (19.48, 23.76)	
**NY**	32.58 (29.68, 35.62)	34.31 (31.89, 36.81)	33.10 (30.97, 35.30)		36.99 (34.68, 39.37)	
**OH**	33.22 (30.43, 36.13)	39.70 (36.65, 42.84)	32.51 (29.78, 35.37)		37.92 (34.93, 41.00)	
**OK**	41.16 (38.28, 44.10)	36.62 (33.89, 39.43)	33.80 (30.99, 36.73)		30.31 (27.96, 32.77)	40.64 (37.97, 43.36)
**OR**	21.48 (19.96, 23.08)	19.68 (18.27, 21.18)	20.19 (18.76, 21.70)		20.77 (19.24, 22.39)	
**PA**	26.99 (25.11, 28.96)	26.44 (24.52, 28.45)	26.40 (24.48, 28.42)	27.07 (25.17, 29.05)	26.43 (24.86, 28.05)	26.68 (25.01, 28.42)
**RI**	26.16 (24.01, 28.42)	27.96 (25.61, 30.45)	25.67 (23.42, 28.05)			
**SC**	34.30 (31.85, 36.83)	36.01 (33.50, 38.59)	36.37 (33.79, 39.03)	35.00 (32.59, 37.49)	31.33 (29.12, 33.63)	37.39 (34.90, 39.95)
**SD**	28.99 (26.68, 31.41)	27.14 (24.89, 29.51)	29.69 (27.46, 32.03)		30.70 (28.37, 33.15)	38.44 (35.94, 41.01)
**TN**	38.50 (36.46, 40.58)	38.65 (36.63, 40.72)	37.86 (35.79, 39.97)	39.17 (37.25, 41.12)	39.68 (37.72, 41.66)	
**TX**	28.71 (26.12, 31.45)	27.04 (24.36, 29.90)	27.43 (25.38, 29.57)		27.80 (25.20, 30.55)	
**UT**	23.23 (21.08, 25.52)	20.73 (18.72, 22.91)	21.77 (19.70, 23.99)		20.94 (18.91, 23.12)	
**VA**	26.16 (23.90, 28.55)	25.23 (22.89, 27.72)	25.87 (23.54, 28.35)	27.06 (24.76, 29.49)	22.91 (20.67, 25.31)	27.08 (24.82, 29.46)
**VT**	24.95 (22.14, 27.99)	26.48 (24.09, 29.01)	25.71 (23.66, 27.88)		23.27 (21.49, 25.15)	
**WA**	19.92 (18.10, 21.88)	20.58 (18.75, 22.54)	18.63 (17.03, 20.34)		18.17 (16.80, 19.62)	
**WI**	24.68 (22.16, 27.40)	25.01 (22.54, 27.67)	22.87 (20.61, 25.29)		25.94 (23.36, 28.71)	
**WV**	39.55 (37.38, 41.77)	42.21 (39.98, 44.48)	40.82 (38.58, 43.11)		45.32 (43.14, 47.52)	
**WY**					20.91 (18.51, 23.55)	23.66 (21.92, 25.50)

**Table 4:** Prevalence (%) of leisure-time physical inactivity in U.S. states, 1996–2001.

State	1996	1997	1998	1999	2000	2001
**AK**	25.39 (22.20, 28.87)		23.57 (21.07, 26.27)		20.05 (17.74, 22.56)	20.99 (18.95, 23.19)
**AL**	32.45 (30.24, 34.74)		29.74 (27.58, 32.00)		31.64 (29.37, 34.00)	31.19 (29.29, 33.15)
**AR**	37.48 (34.85, 40.17)		35.74 (33.78, 37.74)		28.03 (26.27, 29.86)	31.46 (29.58, 33.42)
**AZ**	33.32 (30.45, 36.33)	38.99 (36.10, 41.95)	51.27 (47.79, 54.74)		34.08 (30.77, 37.55)	21.94 (19.86, 24.17)
**CA**	23.59 (22.08, 25.16)		25.54 (24.03, 27.12)		26.47 (24.75, 28.27)	26.56 (24.83, 28.36)
**CO**	20.15 (18.11, 22.36)		21.24 (19.11, 23.53)		19.80 (17.79, 21.96)	19.11 (17.25, 21.12)
**CT**	25.46 (23.29, 27.75)		27.07 (25.15, 29.09)		25.27 (23.74, 26.87)	24.00 (22.86, 25.18)
**DC**	30.10 (27.25, 33.11)		38.07 (35.13, 41.11)		20.79 (18.64, 23.13)	24.20 (21.83, 26.74)
**DE**	36.16 (33.75, 38.64)		35.40 (32.84, 38.04)		27.98 (25.82, 30.25)	25.65 (23.87, 27.52)
**FL**	27.10 (25.48, 28.78)		31.18 (29.63, 32.77)		28.80 (27.37, 30.27)	27.76 (26.25, 29.32)
**GA**	51.28 (48.94, 53.63)		29.56 (27.34, 31.89)	25.87 (23.79, 28.07)	29.02 (27.32, 30.79)	27.26 (25.66, 28.91)
**HI**	21.01 (18.99, 23.19)		17.99 (15.95, 20.22)	25.45 (22.95, 28.12)	23.22 (21.78, 24.73)	19.00 (17.48, 20.61)
**IA**	26.95 (25.38, 28.58)	25.65 (24.02, 27.34)	26.68 (25.03, 28.41)		27.33 (25.69, 29.04)	25.94 (24.34, 27.59)
**ID**	20.53 (18.83, 22.35)		20.40 (19.08, 21.77)		19.81 (18.58, 21.11)	21.09 (19.69, 22.57)
**IL**	24.82 (22.45, 27.37)	28.29 (25.64, 31.10)	27.10 (24.56, 29.79)	29.80 (27.06, 32.70)	30.89 (28.48, 33.41)	26.53 (24.96, 28.17)
**IN**	31.01 (28.92, 33.17)		27.09 (25.19, 29.08)		25.23 (23.51, 27.04)	26.33 (24.84, 27.88)
**KS**	36.34 (34.07, 38.68)		38.20 (36.42, 40.02)		30.37 (28.80, 32.00)	26.73 (25.33, 28.17)
**KY**	45.49 (43.59, 47.42)	37.87 (35.25, 40.55)	42.63 (40.84, 44.44)		41.13 (39.47, 42.81)	33.51 (32.06, 35.00)
**LA**	34.93 (32.34, 37.61)		32.30 (29.82, 34.88)		36.22 (34.71, 37.77)	35.62 (34.14, 37.13)
**MA**	23.03 (20.87, 25.35)		25.40 (23.86, 27.00)		24.65 (23.53, 25.81)	22.86 (21.82, 23.93)
**MD**	33.69 (31.84, 35.60)		20.24 (18.44, 22.17)		24.22 (22.68, 25.84)	24.16 (22.58, 25.82)
**ME**	34.01 (31.51, 36.59)		27.72 (25.28, 30.29)		27.14 (25.02, 29.37)	23.35 (21.58, 25.22)
**MI**	23.17 (21.37, 25.07)		21.36 (19.68, 23.15)	24.48 (22.65, 26.40)	22.90 (21.17, 24.73)	23.38 (21.90, 24.94)
**MN**	23.61 (22.25, 25.03)		25.46 (24.05, 26.93)		24.80 (22.95, 26.75)	17.13 (15.84, 18.50)
**MO**	30.24 (27.76, 32.85)		27.90 (26.07, 29.81)		28.85 (27.08, 30.69)	27.44 (25.68, 29.27)
**MS**	39.56 (36.72, 42.48)		33.84 (31.65, 36.11)		33.37 (31.05, 35.77)	33.43 (31.56, 35.36)
**MT**	21.13 (19.16, 23.24)		25.22 (23.09, 27.47)		23.29 (21.34, 25.37)	21.88 (20.08, 23.78)
**NC**	40.69 (38.59, 42.82)		27.82 (25.55, 30.22)		30.54 (28.69, 32.44)	26.40 (24.75, 28.12)
**ND**	33.86 (31.50, 36.31)		33.13 (30.76, 35.59)		24.28 (22.24, 26.45)	23.24 (21.49, 25.09)
**NE**	22.90 (20.91, 25.01)		26.03 (24.18, 27.97)	28.47 (26.61, 30.41)	29.28 (27.45, 31.18)	30.95 (29.18, 32.78)
**NH**	25.56 (23.04, 28.26)		24.80 (22.40, 27.37)		26.59 (24.36, 28.94)	19.52 (18.18, 20.93)
**NJ**	26.33 (24.57, 28.18)	30.17 (28.03, 32.41)	32.54 (30.37, 34.78)		28.70 (27.01, 30.44)	26.54 (25.08, 28.05)
**NM**	27.65 (24.29, 31.29)		23.02 (21.49, 24.63)	23.01 (21.46, 24.65)	24.43 (22.81, 26.12)	25.92 (24.30, 27.61)
**NV**	22.78 (20.15, 25.63)		24.04 (21.05, 27.31)		24.92 (22.26, 27.80)	22.60 (20.35, 25.01)
**NY**	30.39 (28.85, 31.98)	35.96 (34.13, 37.83)	31.00 (28.95, 33.13)		29.33 (27.46, 31.26)	28.90 (27.16, 30.71)
**OH**	42.48 (39.69, 45.33)	41.82 (39.43, 44.25)	29.92 (27.68, 32.27)	35.07 (32.32, 37.92)	31.42 (29.15, 33.78)	26.24 (24.48, 28.08)
**OK**	38.33 (35.86, 40.86)	33.71 (31.04, 36.47)	42.98 (40.79, 45.20)	47.58 (45.47, 49.69)	34.40 (32.64, 36.20)	32.78 (31.01, 34.59)
**OR**	19.55 (18.04, 21.16)		18.85 (16.92, 20.93)		20.04 (18.61, 21.55)	20.75 (19.06, 22.56)
**PA**	26.27 (24.68, 27.93)		32.73 (30.99, 34.51)		22.99 (21.43, 24.62)	24.65 (23.08, 26.28)
**RI**	26.70 (24.50, 29.02)		29.92 (28.25, 31.65)		27.50 (25.78, 29.30)	24.83 (23.30, 26.42)
**SC**	29.77 (27.25, 32.42)	33.61 (31.46, 35.83)	33.71 (31.82, 35.65)		28.02 (26.26, 29.85)	26.50 (24.74, 28.33)
**SD**	34.69 (32.47, 36.99)		33.33 (31.00, 35.73)		26.63 (25.28, 28.03)	25.32 (24.01, 26.68)
**TN**	40.71 (38.75, 42.70)	33.13 (30.39, 36.00)	35.78 (33.79, 37.82)	32.17 (30.24, 34.17)	32.72 (30.84, 34.66)	35.08 (33.07, 37.14)
**TX**	27.87 (25.57, 30.28)		27.94 (26.45, 29.47)		28.55 (27.11, 30.03)	27.09 (25.78, 28.44)
**UT**	17.05 (15.39, 18.84)		17.10 (15.34, 19.02)	17.36 (15.62, 19.25)	15.53 (13.92, 17.29)	16.53 (15.05, 18.11)
**VA**	29.29 (26.88, 31.81)	24.35 (22.12, 26.73)	24.70 (22.71, 26.80)	22.02 (20.25, 23.90)	24.90 (22.80, 27.12)	23.22 (21.52, 25.01)
**VT**	21.48 (19.69, 23.39)		25.89 (24.10, 27.77)		23.16 (21.61, 24.78)	20.46 (19.13, 21.86)
**WA**	19.07 (17.65, 20.57)		17.57 (16.21, 19.02)		16.86 (15.55, 18.26)	17.11 (15.88, 18.41)
**WI**	22.14 (20.03, 24.41)		23.47 (21.38, 25.70)		22.14 (20.44, 23.93)	20.74 (19.21, 22.36)
**WV**	42.74 (40.47, 45.04)		43.70 (41.46, 45.97)		33.59 (31.55, 35.70)	31.63 (29.82, 33.50)
**WY**	20.28 (18.64, 22.02)	21.61 (19.38, 24.01)	21.05 (19.16, 23.07)		22.62 (20.80, 24.55)	21.25 (19.68, 22.91)

**Table 5:** Prevalence (%) of leisure-time physical inactivity in U.S. states, 2002–2007.

State	2002	2003	2004	2005	2006	2007
**AK**	22.39 (19.88, 25.12)	19.10 (17.12, 21.27)	20.42 (18.28, 22.75)	21.49 (19.36, 23.77)	21.50 (19.16, 24.04)	20.02 (17.75, 22.50)
**AL**	27.33 (25.49, 29.25)	29.95 (28.13, 31.84)	29.68 (27.96, 31.46)	29.70 (27.77, 31.70)	29.10 (27.07, 31.22)	29.87 (28.29, 31.50)
**AR**	27.44 (25.88, 29.06)	29.21 (27.65, 30.82)	26.53 (25.00, 28.12)	30.61 (29.11, 32.15)	28.83 (27.39, 30.32)	28.02 (26.56, 29.53)
**AZ**	22.70 (20.50, 25.06)	21.06 (18.99, 23.30)	24.30 (21.85, 26.94)	22.64 (20.33, 25.13)	22.44 (20.23, 24.82)	22.42 (20.17, 24.85)
**CA**	22.75 (21.14, 24.45)	22.34 (20.81, 23.94)	22.79 (21.27, 24.38)	23.93 (22.49, 25.43)	22.99 (21.51, 24.54)	23.10 (21.62, 24.65)
**CO**	19.28 (17.81, 20.84)	16.87 (15.55, 18.26)	18.71 (17.36, 20.14)	17.27 (16.15, 18.44)	17.31 (16.17, 18.51)	17.29 (16.39, 18.23)
**CT**	21.95 (20.52, 23.45)	20.98 (19.72, 22.30)	18.85 (17.64, 20.12)	21.18 (19.82, 22.61)	19.78 (18.73, 20.88)	19.70 (18.51, 20.95)
**DC**	20.70 (18.58, 22.98)	22.78 (20.36, 25.39)	22.27 (20.34, 24.32)	22.37 (20.61, 24.24)	22.02 (20.33, 23.80)	21.31 (19.63, 23.11)
**DE**	27.08 (25.14, 29.11)	26.55 (24.74, 28.45)	21.85 (20.18, 23.62)	23.41 (21.75, 25.16)	21.57 (19.90, 23.33)	22.20 (20.44, 24.06)
**FL**	27.92 (26.54, 29.34)	27.88 (26.00, 29.84)	23.75 (22.33, 25.23)	26.90 (25.50, 28.36)	25.08 (23.86, 26.33)	25.43 (24.38, 26.51)
**GA**	25.61 (24.01, 27.27)	24.56 (23.21, 25.95)	25.87 (24.27, 27.53)	27.24 (25.62, 28.92)	24.74 (23.38, 26.14)	24.59 (23.21, 26.02)
**HI**	16.04 (14.87, 17.29)	18.31 (16.87, 19.84)		19.43 (18.12, 20.81)	19.30 (18.00, 20.67)	17.96 (16.70, 19.30)
**IA**	21.77 (20.28, 23.33)	22.70 (21.36, 24.09)	21.34 (20.05, 22.70)	24.71 (23.28, 26.19)	22.34 (21.04, 23.70)	22.03 (20.68, 23.43)
**ID**	19.39 (18.13, 20.71)	18.68 (17.41, 20.02)	19.10 (17.85, 20.42)	21.66 (20.25, 23.14)	20.71 (19.32, 22.17)	19.59 (18.23, 21.03)
**IL**	28.68 (27.17, 30.25)	25.69 (24.32, 27.12)	24.87 (23.28, 26.53)	25.65 (24.13, 27.24)	22.41 (20.95, 23.95)	22.99 (21.51, 24.53)
**IN**	27.51 (26.21, 28.85)	26.20 (24.92, 27.52)	25.25 (24.05, 26.49)	27.00 (25.67, 28.37)	25.33 (24.05, 26.66)	24.27 (22.84, 25.77)
**KS**	22.47 (21.14, 23.86)	25.95 (24.51, 27.45)	23.25 (22.23, 24.30)	24.32 (23.20, 25.47)	22.56 (21.46, 23.70)	23.01 (21.86, 24.21)
**KY**	26.64 (25.12, 28.22)	30.55 (29.03, 32.10)	29.83 (28.15, 31.55)	31.53 (29.93, 33.18)	30.34 (28.65, 32.07)	30.15 (28.54, 31.81)
**LA**	33.43 (31.89, 35.00)	30.59 (29.11, 32.11)	29.77 (28.58, 30.99)	33.39 (31.38, 35.46)	31.10 (29.71, 32.52)	30.07 (28.61, 31.57)
**MA**	20.81 (19.68, 21.98)	21.75 (20.59, 22.96)	20.01 (18.87, 21.21)	23.37 (22.15, 24.65)	21.16 (20.08, 22.27)	21.08 (20.25, 21.92)
**MD**	23.07 (21.48, 24.75)	21.29 (19.73, 22.93)	21.79 (20.25, 23.41)	22.90 (21.76, 24.07)	23.02 (21.74, 24.34)	23.11 (21.80, 24.49)
**ME**	25.82 (23.93, 27.81)	20.65 (18.93, 22.49)	21.50 (19.94, 23.14)	22.39 (20.89, 23.96)	20.81 (19.38, 22.32)	20.32 (19.10, 21.61)
**MI**	23.93 (22.54, 25.38)	21.77 (20.22, 23.40)	22.20 (20.83, 23.63)	22.42 (21.53, 23.34)	22.84 (21.51, 24.23)	20.76 (19.52, 22.06)
**MN**	16.24 (15.05, 17.49)	15.04 (13.83, 16.34)	15.88 (14.70, 17.12)	16.21 (14.65, 17.90)	14.19 (12.94, 15.53)	16.74 (15.31, 18.28)
**MO**	26.58 (24.90, 28.33)	24.12 (22.42, 25.91)	24.78 (23.20, 26.43)	25.44 (23.58, 27.39)	23.19 (21.50, 24.97)	25.60 (23.90, 27.38)
**MS**	32.46 (30.74, 34.23)	30.36 (28.80, 31.97)	31.37 (29.88, 32.91)	32.48 (30.77, 34.25)	31.16 (29.63, 32.72)	31.87 (30.42, 33.36)
**MT**	19.24 (17.74, 20.83)	20.19 (18.60, 21.87)	18.75 (17.42, 20.16)	22.43 (20.84, 24.10)	19.35 (18.13, 20.63)	19.65 (18.38, 20.99)
**NC**	29.49 (27.81, 31.22)	25.04 (23.70, 26.43)	24.78 (23.86, 25.72)	25.61 (24.73, 26.51)	23.89 (23.00, 24.80)	24.28 (23.24, 25.34)
**ND**	21.68 (20.08, 23.36)	23.78 (22.15, 25.50)	21.34 (19.72, 23.06)	23.11 (21.65, 24.64)	21.98 (20.48, 23.56)	22.48 (21.01, 24.03)
**NE**	21.94 (20.49, 23.47)	20.69 (19.44, 22.00)	21.52 (20.36, 22.73)	23.78 (22.51, 25.09)	21.00 (19.79, 22.26)	22.14 (20.72, 23.63)
**NH**	19.88 (18.63, 21.19)	19.99 (18.71, 21.33)	18.57 (17.39, 19.82)	21.64 (20.41, 22.93)	19.56 (18.37, 20.80)	19.14 (17.94, 20.41)
**NJ**	26.01 (23.69, 28.48)	26.93 (25.92, 27.96)	25.73 (24.75, 26.74)	29.23 (28.15, 30.33)	27.02 (25.93, 28.14)	26.00 (24.47, 27.59)
**NM**	23.09 (21.64, 24.62)	22.92 (21.60, 24.29)	21.10 (19.87, 22.37)	23.29 (21.91, 24.73)	22.58 (21.27, 23.95)	21.68 (20.35, 23.07)
**NV**	24.79 (22.43, 27.31)	24.74 (22.46, 27.17)	24.29 (22.03, 26.70)	26.81 (24.37, 29.41)	27.08 (24.84, 29.44)	24.35 (22.33, 26.50)
**NY**	24.97 (23.45, 26.55)	27.05 (25.61, 28.55)	26.57 (25.14, 28.05)	27.19 (25.87, 28.55)	26.04 (24.52, 27.61)	24.34 (22.91, 25.84)
**OH**	25.29 (23.67, 26.99)	26.50 (24.75, 28.32)	23.01 (21.15, 24.98)	25.55 (23.96, 27.21)	24.48 (22.36, 26.74)	24.37 (23.27, 25.50)
**OK**	30.63 (29.34, 31.96)	30.43 (29.23, 31.67)	27.74 (26.48, 29.03)	30.63 (29.28, 32.01)	29.81 (28.49, 31.18)	29.62 (28.31, 30.97)
**OR**	17.78 (16.30, 19.37)	18.88 (17.50, 20.35)	17.16 (15.97, 18.42)	18.62 (17.79, 19.49)	16.39 (15.15, 17.71)	17.28 (15.97, 18.66)
**PA**	24.41 (23.44, 25.40)	22.58 (21.09, 24.15)	24.38 (23.13, 25.68)	25.88 (24.75, 27.05)	22.95 (21.59, 24.37)	23.26 (22.01, 24.55)
**RI**	24.68 (23.12, 26.30)	23.34 (21.83, 24.91)	24.24 (22.62, 25.93)	25.90 (24.22, 27.66)	24.80 (23.19, 26.49)	23.40 (21.76, 25.12)
**SC**	24.62 (22.95, 26.37)	23.27 (22.01, 24.58)	23.91 (22.71, 25.14)	26.30 (25.15, 27.49)	24.32 (23.14, 25.55)	24.79 (23.62, 26.00)
**SD**	23.78 (22.36, 25.26)	21.66 (20.41, 22.97)	19.02 (17.87, 20.21)	22.40 (21.18, 23.67)	23.96 (22.52, 25.46)	22.52 (21.19, 23.91)
**TN**	33.52 (31.61, 35.48)	29.76 (27.76, 31.84)	29.71 (27.77, 31.74)	33.07 (31.10, 35.10)	28.70 (26.89, 30.59)	31.43 (29.53, 33.40)
**TX**	29.30 (27.91, 30.72)	27.58 (26.26, 28.95)	26.13 (24.78, 27.53)	27.37 (25.98, 28.80)	28.34 (26.38, 30.40)	28.36 (27.32, 29.43)
**UT**	18.89 (17.30, 20.59)	17.34 (15.81, 18.98)	16.94 (15.71, 18.24)	18.52 (17.21, 19.90)	19.39 (17.93, 20.94)	19.48 (18.03, 21.01)
**VA**	24.50 (22.91, 26.16)	22.06 (20.66, 23.51)	21.90 (20.41, 23.46)	21.39 (19.99, 22.86)	21.70 (20.00, 23.51)	21.58 (20.05, 23.19)
**VT**	18.21 (16.97, 19.52)	18.71 (17.43, 20.07)	18.16 (17.12, 19.24)	19.19 (18.09, 20.34)	17.86 (16.84, 18.94)	18.29 (17.21, 19.42)
**WA**	15.00 (13.75, 16.33)	17.73 (17.04, 18.44)	17.23 (16.54, 17.95)	17.39 (16.74, 18.06)	17.27 (16.58, 17.97)	17.59 (16.95, 18.26)
**WI**	20.06 (18.71, 21.49)	18.81 (17.42, 20.29)	18.41 (17.10, 19.80)	18.65 (17.30, 20.06)	19.36 (17.89, 20.93)	19.48 (18.14, 20.90)
**WV**	28.40 (26.69, 30.16)	28.01 (26.34, 29.74)	24.56 (22.97, 26.22)	28.56 (26.85, 30.33)	25.60 (23.96, 27.30)	28.12 (26.55, 29.74)
**WY**	20.34 (18.85, 21.92)	21.11 (19.72, 22.57)	20.09 (18.75, 21.50)	21.98 (20.69, 23.33)	21.82 (20.50, 23.19)	21.72 (20.40, 23.09)

**Table 6:** Prevalence (%) of leisure-time physical inactivity in U.S. states, 2008–2013.

State	2008	2009	2010	2011	2012	2013
**AK**	24.07 (21.60, 26.73)	22.42 (19.96, 25.08)	21.98 (19.15, 25.11)	22.00 (20.14, 23.98)	18.53 (16.95, 20.21)	21.92 (20.15, 23.80)
**AL**	29.52 (27.87, 31.22)	31.10 (29.42, 32.83)	31.11 (29.59, 32.67)	32.57 (31.06, 34.13)	27.25 (25.91, 28.63)	31.12 (29.41, 32.89)
**AR**	29.75 (28.08, 31.48)	29.78 (27.72, 31.93)	29.80 (27.62, 32.08)	30.88 (28.85, 32.98)	31.61 (29.88, 33.38)	34.03 (32.09, 36.04)
**AZ**	23.06 (20.73, 25.57)	18.86 (17.11, 20.75)	20.82 (18.99, 22.77)	24.10 (22.01, 26.31)	22.27 (20.69, 23.94)	24.96 (22.41, 27.69)
**CA**	23.33 (22.25, 24.45)	22.10 (21.22, 23.00)	20.42 (19.61, 21.26)	19.13 (18.28, 20.00)	19.19 (18.23, 20.19)	21.00 (19.86, 22.19)
**CO**	18.91 (17.98, 19.89)	17.67 (16.67, 18.72)	18.17 (17.09, 19.32)	16.49 (15.54, 17.48)	17.04 (16.11, 18.01)	17.47 (16.57, 18.42)
**CT**	22.51 (21.06, 24.02)	21.64 (20.24, 23.12)	20.66 (19.27, 22.12)	25.31 (23.81, 26.89)	22.10 (20.87, 23.39)	24.12 (22.60, 25.71)
**DC**	21.20 (19.54, 22.95)	19.70 (18.07, 21.44)	19.96 (18.32, 21.70)	19.75 (18.03, 21.60)	17.46 (15.71, 19.36)	19.06 (17.11, 21.17)
**DE**	24.11 (22.18, 26.14)	21.82 (20.15, 23.59)	23.95 (22.21, 25.79)	26.96 (25.18, 28.81)	23.54 (21.98, 25.17)	26.85 (25.16, 28.61)
**FL**	25.93 (24.35, 27.58)	23.63 (22.26, 25.05)	24.02 (23.00, 25.08)	26.94 (25.67, 28.25)	23.33 (21.87, 24.85)	27.22 (26.04, 28.44)
**GA**	23.11 (21.59, 24.69)	24.21 (22.53, 25.98)	25.11 (23.53, 26.75)	26.76 (25.41, 28.15)	23.66 (22.23, 25.14)	26.78 (25.36, 28.25)
**HI**	19.51 (18.14, 20.96)	19.62 (18.29, 21.04)	19.18 (17.80, 20.64)	21.26 (19.79, 22.80)	18.66 (17.29, 20.11)	21.09 (19.65, 22.61)
**IA**	25.06 (23.70, 26.47)	24.18 (22.78, 25.64)	24.74 (23.28, 26.26)	25.87 (24.59, 27.19)	22.94 (21.77, 24.15)	27.68 (26.28, 29.13)
**ID**	21.09 (19.65, 22.60)	21.05 (19.60, 22.58)	19.95 (18.69, 21.27)	21.36 (19.74, 23.08)	20.13 (18.41, 21.98)	23.31 (21.59, 25.13)
**IL**	27.95 (26.29, 29.68)	23.59 (22.13, 25.12)	25.71 (24.05, 27.46)	25.17 (23.51, 26.91)	21.80 (20.26, 23.41)	24.22 (22.52, 25.99)
**IN**	27.69 (25.82, 29.64)	27.21 (25.92, 28.55)	26.45 (25.14, 27.80)	29.26 (27.92, 30.64)	25.83 (24.64, 27.06)	30.41 (29.15, 31.70)
**KS**	25.54 (24.32, 26.79)	23.18 (22.37, 24.00)	23.99 (22.80, 25.21)	26.83 (26.03, 27.64)	22.85 (21.82, 23.91)	25.96 (25.21, 26.71)
**KY**	30.54 (28.99, 32.13)	29.74 (28.17, 31.36)	29.26 (27.66, 30.92)	29.35 (27.95, 30.79)	29.71 (28.42, 31.02)	29.52 (28.12, 30.95)
**LA**	29.80 (28.30, 31.35)	28.59 (27.26, 29.96)	30.16 (28.63, 31.73)	33.78 (32.33, 35.25)	29.98 (28.46, 31.55)	31.84 (29.75, 34.01)
**MA**	22.11 (21.22, 23.04)	20.96 (19.92, 22.05)	20.60 (19.64, 21.59)	23.50 (22.54, 24.49)	19.78 (18.97, 20.62)	22.78 (21.61, 23.99)
**MD**	23.99 (22.72, 25.31)	23.81 (22.48, 25.18)	23.12 (21.86, 24.43)	26.12 (24.75, 27.54)	22.95 (21.70, 24.25)	24.51 (23.28, 25.77)
**ME**	22.84 (21.58, 24.14)	21.22 (20.11, 22.37)	22.44 (21.22, 23.70)	23.02 (22.07, 24.00)	20.93 (19.94, 21.96)	22.75 (21.46, 24.09)
**MI**	25.09 (23.91, 26.32)	23.54 (22.40, 24.72)	23.62 (22.38, 24.91)	23.59 (22.41, 24.81)	23.28 (22.14, 24.46)	23.82 (22.75, 24.93)
**MN**	18.08 (16.65, 19.61)	15.76 (14.44, 17.16)	19.12 (17.76, 20.57)	21.83 (20.85, 22.84)	17.60 (16.69, 18.54)	22.29 (20.94, 23.71)
**MO**	27.57 (25.87, 29.33)	26.61 (24.79, 28.50)	27.13 (25.20, 29.15)	28.47 (26.89, 30.10)	24.73 (23.29, 26.24)	27.66 (26.02, 29.35)
**MS**	32.55 (31.09, 34.04)	32.37 (31.06, 33.71)	33.03 (31.46, 34.64)	35.97 (34.56, 37.42)	30.83 (29.37, 32.33)	37.60 (35.88, 39.35)
**MT**	23.07 (21.65, 24.55)	22.07 (20.75, 23.45)	21.55 (20.10, 23.08)	24.36 (23.08, 25.69)	20.56 (19.49, 21.67)	21.79 (20.60, 23.03)
**NC**	24.67 (23.70, 25.67)	26.43 (25.10, 27.81)	25.78 (24.53, 27.07)	26.70 (25.38, 28.06)	24.87 (23.89, 25.88)	26.05 (24.76, 27.39)
**ND**	25.51 (23.93, 27.16)	26.73 (25.07, 28.45)	24.77 (23.05, 26.58)	27.00 (25.45, 28.60)	23.76 (22.20, 25.39)	26.99 (25.50, 28.53)
**NE**	24.68 (23.36, 26.05)	24.19 (22.85, 25.59)	24.71 (23.37, 26.11)	26.28 (25.48, 27.11)	21.00 (20.20, 21.84)	24.70 (23.57, 25.86)
**NH**	21.49 (20.25, 22.79)	21.23 (19.81, 22.72)	20.04 (18.72, 21.42)	22.52 (21.10, 24.02)	19.98 (18.73, 21.30)	21.80 (20.33, 23.34)
**NJ**	26.95 (25.75, 28.18)	26.22 (25.03, 27.45)	26.53 (25.33, 27.78)	26.45 (25.37, 27.56)	25.01 (24.03, 26.02)	25.92 (24.69, 27.19)
**NM**	23.93 (22.33, 25.61)	22.44 (21.12, 23.82)	21.60 (20.19, 23.08)	25.26 (24.03, 26.52)	21.76 (20.68, 22.87)	23.86 (22.53, 25.25)
**NV**	27.65 (25.64, 29.75)	24.45 (22.12, 26.94)	22.91 (20.65, 25.34)	24.09 (21.98, 26.33)	21.23 (19.56, 23.00)	23.30 (21.11, 25.64)
**NY**	26.30 (24.97, 27.68)	26.42 (24.92, 27.99)	23.88 (22.71, 25.08)	26.24 (24.86, 27.68)	24.67 (23.11, 26.30)	26.03 (24.71, 27.39)
**OH**	26.03 (24.90, 27.18)	26.35 (25.04, 27.69)	26.09 (24.81, 27.41)	27.03 (25.78, 28.31)	25.28 (24.26, 26.33)	27.64 (26.37, 28.94)
**OK**	31.49 (30.16, 32.85)	31.37 (30.01, 32.76)	29.96 (28.62, 31.33)	31.20 (29.83, 32.60)	28.29 (27.03, 29.59)	32.42 (31.05, 33.82)
**OR**	18.96 (17.52, 20.49)	17.73 (16.24, 19.33)	17.51 (16.08, 19.04)	19.74 (18.27, 21.30)	16.30 (15.01, 17.69)	18.14 (16.65, 19.72)
**PA**	25.75 (24.54, 26.99)	25.73 (24.46, 27.05)	25.74 (24.63, 26.88)	26.29 (25.10, 27.50)	23.46 (22.58, 24.35)	25.18 (24.04, 26.36)
**RI**	24.26 (22.65, 25.96)	24.87 (23.40, 26.39)	24.06 (22.62, 25.57)	26.19 (24.72, 27.71)	23.55 (22.05, 25.12)	26.08 (24.51, 27.71)
**SC**	27.24 (25.75, 28.79)	26.17 (24.74, 27.65)	27.86 (26.17, 29.61)	27.21 (25.96, 28.50)	25.10 (23.99, 26.23)	26.36 (25.10, 27.66)
**SD**	26.84 (25.32, 28.41)	24.50 (23.04, 26.01)	24.67 (23.17, 26.23)	26.89 (25.02, 28.84)	22.51 (21.02, 24.09)	22.93 (21.20, 24.75)
**TN**	28.93 (26.93, 31.02)	31.00 (29.13, 32.93)	29.95 (28.01, 31.96)	35.17 (32.61, 37.81)	28.62 (27.17, 30.11)	36.92 (35.00, 38.89)
**TX**	28.47 (27.02, 29.97)	27.34 (25.89, 28.84)	26.67 (25.35, 28.03)	27.16 (25.86, 28.49)	27.16 (25.85, 28.52)	29.54 (28.07, 31.06)
**UT**	19.78 (18.35, 21.28)	17.67 (16.62, 18.76)	17.92 (16.84, 19.07)	18.93 (17.99, 19.91)	16.50 (15.62, 17.43)	20.15 (19.19, 21.16)
**VA**	23.55 (21.52, 25.71)	21.96 (20.20, 23.82)	23.40 (21.51, 25.41)	25.04 (23.44, 26.70)	22.53 (21.30, 23.81)	24.95 (23.61, 26.34)
**VT**	19.37 (18.23, 20.56)	20.15 (18.91, 21.44)	17.89 (16.71, 19.14)	20.99 (19.71, 22.33)	17.22 (16.05, 18.44)	19.83 (18.54, 21.18)
**WA**	19.32 (18.59, 20.06)	19.56 (18.77, 20.38)	18.28 (17.47, 19.11)	21.94 (20.80, 23.13)	18.96 (18.11, 19.85)	19.60 (18.49, 20.75)
**WI**	22.01 (20.46, 23.64)	22.05 (20.20, 24.01)	22.91 (21.02, 24.92)	22.60 (20.87, 24.43)	20.33 (18.77, 21.98)	23.25 (21.56, 25.02)
**WV**	31.09 (29.39, 32.84)	33.21 (31.61, 34.85)	32.96 (31.17, 34.80)	35.14 (33.59, 36.73)	30.95 (29.51, 32.43)	30.60 (29.14, 32.09)
**WY**	24.35 (23.19, 25.55)	22.39 (21.00, 23.84)	22.11 (20.72, 23.56)	25.31 (23.79, 26.88)	21.17 (19.59, 22.84)	24.38 (22.81, 26.02)

**Table 7:** Prevalence (%) of leisure-time physical inactivity in U.S. states, 2014–2015.

State	2014	2015
AK	18.82 (17.18, 20.59)	21.50 (19.19, 24.00)
AL	26.92 (25.57, 28.31)	31.36 (29.84, 32.92)
AR	29.89 (27.97, 31.88)	33.72 (31.36, 36.16)
AZ	20.53 (19.41, 21.70)	24.42 (22.89, 26.03)
CA	21.40 (20.21, 22.63)	19.54 (18.57, 20.54)
CO	15.72 (14.88, 16.59)	17.37 (16.31, 18.47)
CT	19.32 (18.02, 20.68)	22.60 (21.38, 23.85)
DC	20.27 (18.07, 22.67)	18.77 (16.33, 21.49)
DE	24.32 (22.44, 26.31)	28.99 (26.82, 31.26)
FL	22.88 (21.68, 24.13)	25.81 (24.42, 27.26)
GA	23.03 (21.60, 24.52)	26.87 (25.00, 28.82)
HI	18.87 (17.57, 20.24)	21.86 (20.46, 23.34)
IA	21.60 (20.43, 22.83)	25.65 (24.11, 27.25)
ID	18.17 (16.65, 19.79)	20.76 (19.19, 22.41)
IL	23.22 (21.63, 24.89)	24.17 (22.62, 25.78)
IN	25.01 (23.91, 26.14)	28.60 (26.80, 30.48)
KS	22.94 (22.06, 23.85)	25.90 (25.13, 26.68)
KY	27.47 (26.07, 28.91)	31.77 (30.08, 33.51)
LA	28.86 (27.48, 30.28)	31.56 (29.70, 33.49)
MA	19.11 (18.07, 20.18)	25.66 (24.27, 27.09)
MD	20.69 (19.34, 22.11)	23.40 (21.77, 25.11)
ME	18.67 (17.56, 19.82)	23.90 (22.59, 25.27)
MI	24.67 (23.42, 25.96)	24.94 (23.72, 26.21)
MN	19.51 (18.74, 20.31)	20.96 (20.12, 21.82)
MO	24.25 (22.72, 25.84)	26.49 (24.97, 28.06)
MS	31.07 (29.07, 33.14)	36.24 (34.36, 38.17)
MT	18.78 (17.48, 20.15)	21.49 (19.94, 23.11)
NC	22.55 (21.39, 23.76)	25.65 (24.31, 27.04)
ND	20.48 (19.06, 21.96)	26.19 (24.48, 27.98)
NE	20.37 (19.51, 21.26)	24.77 (23.70, 25.87)
NH	18.31 (16.93, 19.78)	21.91 (20.45, 23.44)
NJ	22.48 (21.37, 23.64)	26.40 (25.00, 27.84)
NM	22.59 (21.20, 24.03)	22.11 (20.55, 23.76)
NV	21.81 (19.73, 24.04)	24.35 (21.82, 27.06)
NY	25.20 (23.83, 26.62)	28.77 (27.56, 30.00)
OH	24.11 (22.81, 25.47)	26.13 (24.73, 27.59)
OK	27.58 (26.35, 28.84)	32.63 (30.90, 34.41)
OR	15.80 (14.47, 17.23)	18.12 (16.64, 19.70)
PA	22.34 (21.20, 23.52)	27.04 (25.39, 28.75)
RI	21.53 (20.08, 23.05)	27.16 (25.34, 29.05)
SC	24.78 (23.66, 25.95)	26.23 (25.05, 27.45)
SD	20.49 (18.78, 22.31)	20.66 (19.00, 22.44)
TN	26.15 (24.45, 27.92)	30.03 (28.09, 32.03)
TX	27.10 (25.87, 28.37)	29.10 (27.59, 30.65)
UT	16.25 (15.50, 17.04)	19.96 (19.01, 20.96)
VA	23.04 (21.87, 24.25)	24.38 (23.03, 25.77)
VT	18.13 (17.01, 19.30)	21.60 (20.19, 23.09)
WA	17.58 (16.47, 18.75)	18.56 (17.65, 19.49)
WI	20.23 (18.80, 21.73)	20.82 (19.31, 22.43)
WV	28.01 (26.64, 29.43)	30.06 (28.63, 31.51)
WY	21.22 (19.53, 23.02)	25.72 (23.75, 27.78)

*Notes*: BRFSS survey design was incorporated in prevalence estimation. 95% confidence interval is reported in parenthesis.

**Fig 2:**
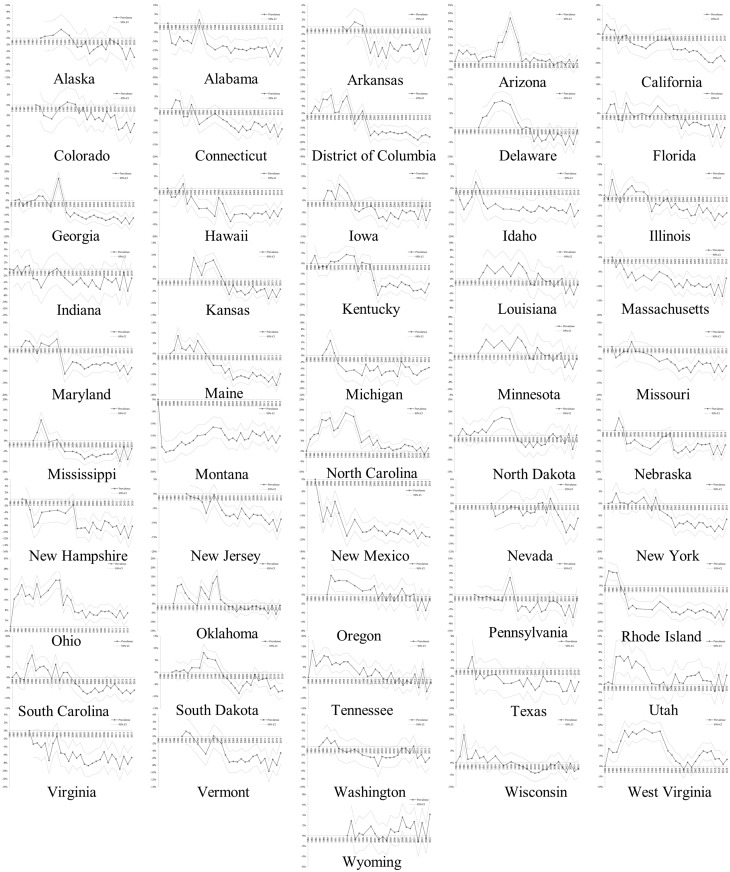
Regression-adjusted changes in the prevalence of leisure-time physical inactivity.

Fig 2 illustrates state-specific regression-adjusted changes in the prevalence of leisure-time physical inactivity over the BRFSS survey period, with the prevalence in the first survey year as the baseline. Despite some year-to-year variations, the adjusted prevalence of leisure-time physical inactivity gradually declined over the survey period in a majority of states. For example, the adjusted prevalence in New Mexico and Rhode Island decreased by 24.0% and 13.4% during 1986–2015, respectively. In contrast, the adjusted prevalence of leisure-time physical inactivity either remained largely unchanged or even increased over the survey period in a few states. For example, the adjusted prevalence of leisure-time physical inactivity in Arizona, North Carolina, North Dakota, Utah, West Virginia, and Wyoming in 2015 was largely identical to the prevalence at the baseline survey year, respectively. The adjusted prevalence in Minnesota and Ohio increased by 6.2% and 6.6% from 1984 to 2015, respectively.

## Discussion

This study estimated the prevalence of leisure-time physical inactivity across U.S. states during 1984–2015 based on data from the BRFSS. The prevalence of leisure-time physical inactivity varied substantially across states and survey years. Despite these year-to-year variations, the adjusted prevalence of leisure-time physical inactivity gradually declined over the survey period in a majority of states, after controlling for sample sociodemographics including sex, age, race/ethnicity, education, marital status, and employment status. Conversely, the adjusted prevalence of leisure-time physical inactivity in several states either remained largely unchanged (e.g., Arizona, North Carolina, North Dakota, Utah, West Virginia, and Wyoming) or even increased (e.g., Minnesota and Ohio) over the study period.

The finding on the overall decline in the prevalence of leisure-time physical inactivity in a majority of states is consistent with previous studies that examined shorter survey periods of the BRFSS [26–28]. Similarly, studies using other national data sources including the National Health Interview Survey and the National Health and Nutrition Examination Survey have found a significant decline in the prevalence of leisure-time physical inactivity and/or an increase in leisure-time physical activity [29,30]. The decrease in leisure-time physical inactivity could partially result from public health campaigns, policies and programs with the aim of promoting active lifestyle in the population. A widespread call to prevent chronic disease through physical activity followed the publication of the *1996 Physical Activity and Health*: *A Report of the Surgeon General* and the *2008 Physical Activity Guidelines for Americans* [2,19]. Professional associations, the CDC, and the National Institute of Health have subsequently published recommendations for regular physical activity [31]. Health care providers, payers, employees, and community groups have also joined the call to promote physical activity during the past decade [31]. In addition, improving health, fitness, and quality of life through daily physical activity is listed as a key goal in the *Healthy People 2020* [32].

A few states did not witness a decline in leisure-time physical inactivity during the past three decades, including Arizona, North Carolina, North Dakota, Utah, West Virginia, Wyoming, Minnesota, and Ohio. Minnesota and Ohio, in particular, saw an increase in leisure-time physical inactivity. Using data from the BRFSS, Dwyer-Lindgren et al (2013) found that an increase in physical activity level was associated with a small but significant decrease in obesity prevalence at the county-level [27]. Two counties in Ohio were placed at the top 10 counties with the largest gains in adult obesity rates from 2001–2009, which coincided with the increased prevalence of physical inactivity in Ohio [27]. It is also possible that efforts to promote physical activity in these states have not been as successful as the rest of the country. This study did not examine why physical activity level has not improved in these states. Future studies should conduct in-depth analysis and case studies to identify the causes and develop effective and targeted programs to improve physical activity in these states.

Despite an overall decrease in leisure-time physical inactivity in a majority of states, a substantial proportion of American adults remain physically inactive. Among the 50 states and District of Columbia, 45 had over a fifth of their adult population without any leisure-time physical activity, and 8 had over 30% without physical activity in 2015. The high prevalence of physical inactivity coincides with the increase in adult obesity rates and a rapidly ageing landscape [33]. A recent study showed an inverse dose-response association between levels of physical activity and multimorbidity in older adults [34]. Given the growth of an ageing population and high burden of obesity and multimorbidity, promoting physical activity in all age groups can be vitally important in chronic disease prevention and healthy ageing in place [30].

Closely monitoring state-level physical inactivity is essential in informing policy and program development that aim to promote physical activity and reduce the burden of chronic disease. Physical activity surveillance has progressed substantially in the U.S. and worldwide during the last few decades, which have provided invaluable guidance for health promotion endeavors at the national and community level [35]. The physical activity maps we created can assist states to design, implement, and evaluate policy efforts to promote physical activity. The federal government agencies can use the maps to allocate resources more efficiently and identify areas that need the most assistance to achieve the *Healthy People 2020* physical activity goal [32].

This paper, to our knowledge, is the first study that estimates and tracks the changes in the prevalence of leisure-time physical inactivity across U.S. states during the past 32 years from 1984 to 2015. The BRFSS was the optimal data source for fulfilling this task given its state-representative sampling frame, uniquely large sample size, and fairly consistent wording on the question about physical activity in a span of over three decades. Despite these strengths, a few study limitations should be noted. The BRFSS adopted a crude measure of leisure-time physical activity without distinguishing types, frequency, and intensity of activities, which is a common limitation in population surveillance surveys. Although refined and more detailed questions on physical activity are available, they have been administered only in the most recent BRFSS surveys and thus less useful for tracking long-term trends. Leisure-time physical activity was based on self-report and subject to recall error and social desirability bias [36]. The physical activity measure pertained to leisure-time activities only and did not cover work-related activities. States gradually adopted the BRFSS over the years so that prevalence estimates in early survey period (e.g., 1980s) were available for some but not all states. Questions on leisure-time physical activity were included in the optional (rather than the core) module in the BRFSS 1993, 1995, 1997, and 1999 surveys, which were only administered in a small number of states. The question wording regarding leisure-time exercise slightly changed from 2001 and onward, which may have influenced our estimates on the state prevalence of physical inactivity. Moreover, the sampling scheme of BRFSS changed in 2011 so that a direct comparison between prevalence estimates before and after 2011 should be done with caution [37], although we did not find major changes in the estimated state prevalence of physical inactivity between 2010 and 2011. The response rate of BRFSS is moderate in comparison to other nationally-representative telephone-based health surveys [38], but still about 50%-60% of individuals did not respond to the survey. Despite that the non-response is accounted for in the BRFSS sampling weights, it compromises statistical precision of estimates, and exposes them to non-response bias. The BRFSS remains the largest national health survey in the U.S. and worldwide [39]; however, the sample size of each state still tends to be less optimal in precisely estimating some state health indicators. As seen in the state estimates of physical inactivity prevalence, its variations denoted by the confidence intervals are large, which to some extent impedes an accurate delineation of the trajectory over time.

## Conclusions

Although the prevalence of leisure-time physical inactivity gradually declined over the past three decades in a majority of U.S. states, the rates of physical inactivity remain substantially high and vary considerably across states. Closely monitoring and tracking physical activity level using the state physical activity maps can help guide policy and program development to promote physical activity and reduce the burden of chronic disease.
